# Determining the optimal maximal and submaximal voluntary contraction tests for normalizing the erector spinae muscles

**DOI:** 10.7717/peerj.7824

**Published:** 2019-10-18

**Authors:** Gemma Biviá-Roig, Juan Francisco Lisón, Daniel Sánchez-Zuriaga

**Affiliations:** 1 Department of Physiotherapy, Faculty of Health Sciences, University CEU-Cardenal Herrera, CEU Universities, Spain; 2 Department of Medicine, Faculty of Health Sciences, University CEU-Cardenal Herrera, CEU Universities, Spain. CIBER of Physiopathology of Obesity and Nutrition CIBERobn, CB06/03 Carlos III Health Institute, Spain; 3 Department of Anatomy and Human Embryology, Faculty of Medicine, Universitat de València, València, Spain

**Keywords:** Erector spinae, Electromyography, Sub-maximum voluntary isometric contraction, Normalization, Maximum voluntary isometric contraction

## Abstract

**Background:**

This study aimed to identify which maximum voluntary isometric contraction (MVIC) and sub-MVIC tests produce the highest activation of the erector spinae muscles and the greatest reduction in inter-individual variability, to put them forward as reference normalization maneuvers for future studies.

**Methods:**

Erector spinae EMG activity was recorded in 38 healthy women during five submaximal and three maximal exercises.

**Results:**

None of the three MVIC tests generated the maximal activation level in all the participants. The maximal activation level was achieved in 68.4% of cases with the test performed on the roman chair in the horizontal position (96.3 ± 7.3; *p* < 0.01). Of the five submaximal maneuvers, the one in the horizontal position on the roman chair produced the highest percentage of activation (61.1 ± 16.7; *p* < 0.01), and one of the lowest inter-individual variability values in the normalized signal of a trunk flexion-extension task.

**Conclusions:**

A modified Sorensen MVIC test in a horizontal position on a roman chair and against resistance produced the highest erector spinae activation, but not in 100% of participants, so the execution of several normalization maneuvers with the trunk at different inclinations should be considered to normalize the erector spinae EMG signal. A modified Sorensen test in a horizontal position without resistance is the submaximal maneuver that produces the highest muscle activation and the greatest reduction in inter-individual variability, and could be considered a good reference test for normalization.

## Introduction

Surface electromyography (EMG) is a non-invasive method that allows the level of muscle activity to be quantified. The electromyographic signal is sensitive to many factors, including electrode placement (Jensen, Fuglsang-Frederiksen & Olesen, 1993), subcutaneous fat thickness (McGill, 1991), skin temperature and impedance (Winkel & Jørgensen, 1991), and electrical signals from adjacent muscles (Hansson et al., 1992). This variability can affect the interpretation of the surface EMG (De Luca, 1997). To allow muscle activity to be compared between different muscles, patients, or varying electrode placements on the same muscle or on different days, the electromyographic signal must first be normalized (Lehman & McGill, 1999). In this procedure the absolute electrical activity values are expressed as a percentage with respect to a reference contraction. Such normalization techniques, when applied correctly, have the added effect to reduce inter-subject variability, which improves the reliability of the measurements (Yang & Winter, 1984).

In the general healthy population, the most commonly used normalization method requires performing a maximum voluntary isometric contraction (MVIC) of the muscles being studied (Lehman & McGill, 1999; Allison, Marshall & Singer, 1993; Yang & Winter, 1984). However, in elderly people (Lehman & McGill, 1999) or symptomatic patients who cannot perform maximal contractions because of pain and muscle inhibition (Bolgla & Uhl, 2007; Veiersted, 1991) or a risk of injury (Veiersted, 1991), the use of MVIC maneuvers has been called into question and submaximal maneuvers are instead recommended as a normalization method (Dankaerts et al., 2004; O’Sullivan et al., 1997).

Some authors have investigated which normalization maneuver produces the highest level of activation for different muscles, including for the shoulder muscles (Dal Maso, Marion & Begon, 2016; Chopp, Fischer & Dickerson, 2010; Boettcher, Ginn & Cathers, 2008), scapula (Castelein et al., 2015), and trunk (Vera-Garcia, Moreside & McGill, 2010). These studies indicate that no single test produces maximal activation in every individual but rather, there may be several maneuvers capable of producing the maximal activation for a given muscle.

At the level of the trunk, the maximal test most commonly used to normalize EMG activation in the erector spinae is extension of the trunk against resistance in the horizontal position, known as the Biering–Sorensen maneuver (McGill, 1991; Vera-Garcia, Moreside & McGill, 2010; Barbado et al., 2012; García-Vaquero et al., 2012). Although some studies on fatigue have shown differences in the degree of activation of the erector spinae during trunk extension in different positions (Champagne, Descarreaux & Lafond, 2008), none have compared different trunk positions to try to standardize MVIC maneuvers.

However, most studies on the erector spinae focus on patients with low back pain (LBP) and so, to avoid the possibility of exacerbating pain, researchers have had to resort to studying submaximal contractions. Different types of submaximal tests for normalizing these muscles have been described in the literature, including the standing position weight-holding test (Lehman, 2002; Callaghan & McGill, 2001) and trunk extension in a horizontal position without resistance (Biviá-Roig, Lisón & Sánchez-Zuriaga, 2018; Biviá-Roig, Lisón & Sánchez-Zuriaga, 2019). One of the main problems caused by using different submaximal normalization maneuvers is the difficulty in subsequently comparing studies. This would require establishing a submaximal reference maneuver capable of achieving sufficiently intense muscle activation for it to be useful in the normalization of a wide variety of tasks.

To shed more light on the maximal and submaximal maneuvers that generate the highest levels of activation for the erector spinae and the greatest reduction in inter-individual variability, we decided to compare their EMG activation by performing a battery of maximal and submaximal tests in different positions on a roman chair and while standing. Our hypothesis was that there would be statistically significant differences between the levels of activation caused by each maneuver. The objective of this study was to identify which MVIC and sub-MVIC tests produce the highest voluntary activation of the trunk muscle extensor muscles and the greatest reduction in inter-individual variability, with the purpose of proposing these as the reference maneuvers for future studies investigating the EMG responses of these muscles.

## Materials & Methods

### Participants

A total of 38 healthy women with no past history of low back pathology participated in the study (age: 32.9 ± 4.9 y; weight: 59.4 ± 8.6 kg; height: 163.8 ± 5.5 cm; BMI 22.1 ± 2.9). All the procedures were conducted in accordance with the principles of the World Medical Association’s Declaration of Helsinki and all participants provided their written informed consent. This research was approved by the local Corporate Ethics Committee for Clinical Research in Primary Care, and the University Ethics Committee on Human Research.

### Instrumentation

Erector spinae EMG activity was recorded by an EMG100C Biopac module (Biopac Systems, Inc., Goleta, CA, USA), using 2 pre-gelled disposable silver-silver chloride (Ag/AgCl) surface disk electrodes (two cm diameter) placed three cm to the right of the L3 spinous process, which was identified through careful palpation by an experienced observer. A reference electrode was placed at the level of the sternal body. The raw EMG signal was band-pass filtered (cutoff frequencies: 10 Hz high pass, 500 Hz low pass) and amplified (input impedance greater than 100 M Ω, common mode rejection ratio of 110 dB at 60 Hz, overall gain of 1,000). EMG signals were A/D converted at a sampling frequency of 1,000 Hz with a 16-bit data acquisition system (model MP150; Biopac Systems Inc.).

### Procedure

All the participants performed a battery of tests comprising five submaximal exercises and three maximal exercises. The women were verbally and visually instructed on the correct performance of the exercises; and each performed two repetitions lasting 5 s separated by two min of rest between each repetition (Konrad, 2005). The order in which the exercises were carried out was randomized in each participant using a computer application. In addition, as a reference task for normalization, the participants performed a trunk flexion-extension movement.

### Test positions

#### Submaximal normalization maneuvers

**Figure 1:**
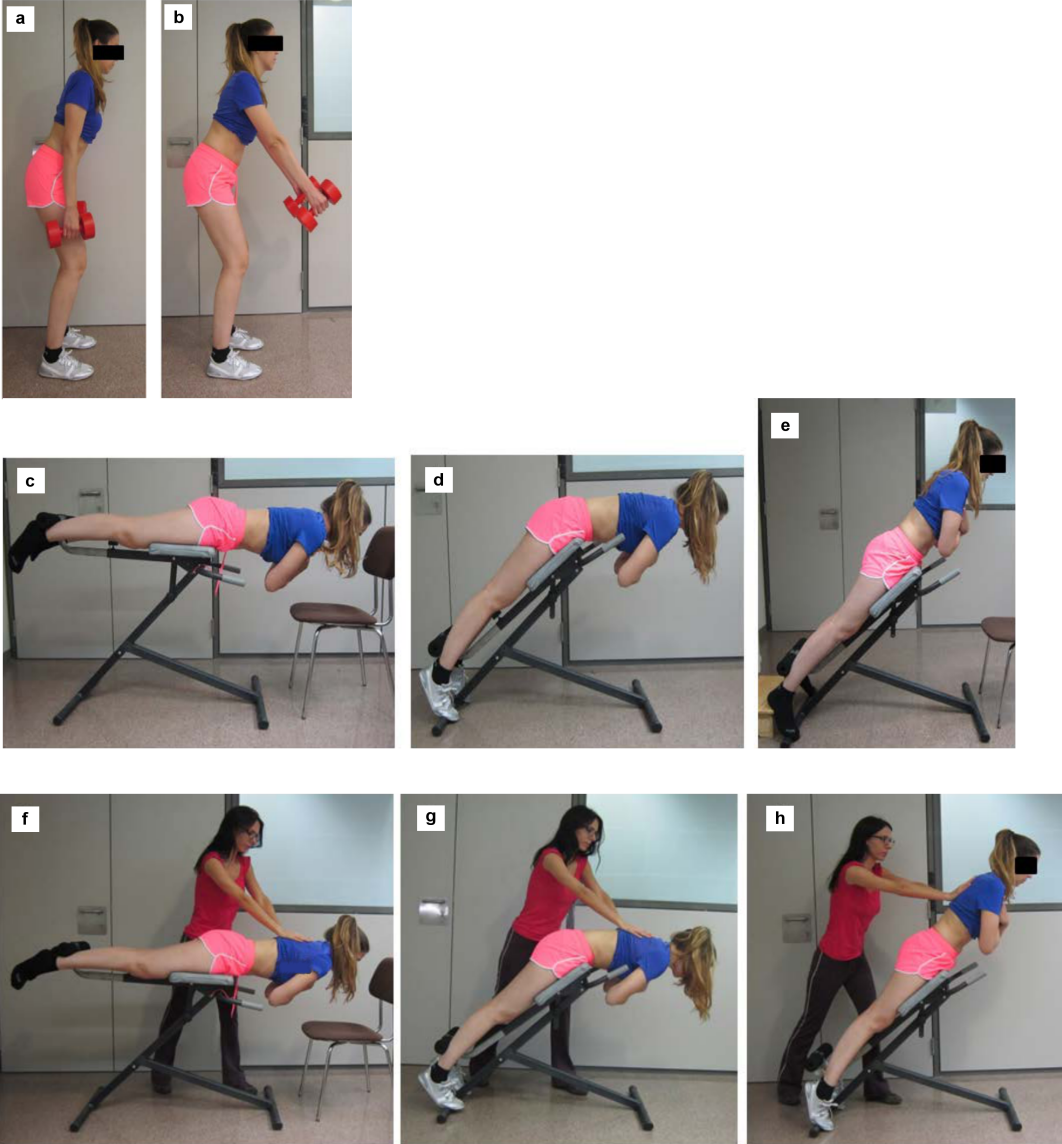
Submaximal and maximal positions. The submaximal standing position weight-holding test (A) with the arms relaxed; (B) with the arms at 45° with respect to the trunk. Submaximal trunk extension tests: (C) horizontal position with respect to the ground; (D) in a 45° position with the hips flexed and the trunk parallel to the ground; (E) in a 45° position with the trunk aligned with the lower limbs. Maximal trunk extension tests: (F) horizontal position with respect to the ground; (G) in a 45° position with the hips flexed and the trunk parallel to the ground; (H) in a 45° position with the trunk aligned with the lower limbs.

The following submaximal maneuvers were performed:

The standing position weight-holding test. This is a variant of the exercises described by Lehman (2002) and Callaghan & McGill (2001). The participants were placed standing facing forward, with 15° of knee flexion and 40° of hip flexion and asked to hold a 3 kg dumbbell in each hand. The exercise was carried out with the arms placed in two different positions: (a) relaxed next to the body; (b) at a 45° angle with respect to the trunk (Fig. 1). In the latter case, a goniometer was used to standardize the position between participants.

A trunk extension test on a roman chair in three different positions (Fig. 1):

(a) With the roman chair placed horizontally with respect to the ground; this exercise is a modification of the Biering–Sorensen test (Biering-Sorensen, 1984).
(b) With the roman chair in a 45° position with respect to the horizontal and with the hips flexed and the trunk resting parallel to the ground.
(c) With the roman chair in a 45° position with respect to the horizontal and with the trunk aligned with the lower limbs.

In all three cases, the participants were placed in a prone position on the roman chair, with the ankles secured, the pelvis supported up to the level of the greater trochanter, and the hands resting on a chair in front of the hyperextension bench. Once placed in each position, the participants were asked to cross their arms over their chest; in the first two positions, they kept their trunk in a horizontal position with respect to the ground, and in the third position, their trunk was oblique and aligned with the lower limbs. Again a goniometer was used to standardize the position of the trunk, the lower limbs and the roman chair between participants.

#### Maximal normalization maneuvers

For the erector spinae MVIC, the Biering–Sorensen exercise was performed on a roman chair in the same three positions already described for the submaximal maneuvers (Fig. 1). In this case, the participant was asked to perform an extension against manual resistance applied by the researcher (Vera-Garcia, Moreside & McGill, 2010).

#### Trunk flexion-extension

From a standing position, the participants performed 5 cycles of trunk flexion-extension movements. Each cycle consisted of maximal trunk flexion from the standing position (4 s), maintenance of maximal flexion (1 s), and extension of the trunk to return to the initial position (4 s).

### Data processing

The EMG signal was rectified and smoothed by calculating its quadratic mean with a window of 0.02 s. Each trial was visually inspected, and the portions of the EMG recording marred with artifacts were excluded from further analyses. Maximum activation values were automatically determined from the artifact-free portions of the recordings by means of a MATLAB 2010a (MathWorks, Inc., Natick, MA, USA) application.

EMG values were subsequently expressed as a percentage of the maximal value of the maneuver in which the highest value was obtained in each participant (Vera-Garcia, Moreside & McGill, 2010).

The maximum activation values of the trunk flexion-extension task for each participant were normalized as a percentage of each of the five submaximal maneuvers, in order to check which one caused a greater reduction of inter-individual variability.

### Statistical analysis

SPSS version 18.0 for Windows (SPSS, Inc., Chicago, IL, USA) was used for all analysis.

To compare the maximal EMG activity of the reference movement (trunk flexion-extension) and the different normalization maneuvers, we carried out a repeated measure analysis of variance (ANOVA), taking the test type/standardization maneuver as an independent variable. To specifically compare each pair of normalization maneuvers, the Bonferroni test was used as a post-hoc test. A *p*-value of 0.05 was considered the threshold for significance in all the comparisons. The *p*-values of post-hoc comparisons are reported as the adjusted *p*-values after the Bonferroni correction, as provided by SPSS.

Inter-individual variability for each submaximal normalization task was measured as the standard deviation of the maximum activation values of trunk flexion-extension, normalized as a percentage of each submaximal task. Plain standard deviation was preferred over other measures, such as the coefficient of variation. The magnitude of the output from different normalization methods can vary considerably, which has the potential to either elevate or reduce the coefficient of variation. Thus, use of the coefficient of variation should be viewed with caution when used to compare the outputs of different normalization methods (Burden, Trew & Baltzopoulos, 2003; Burden, 2010).

## Results

**Figure 2:**
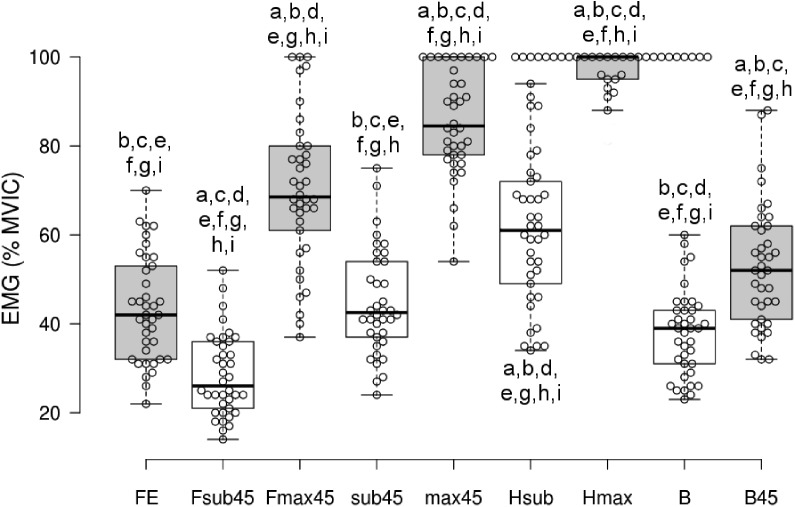
Graph showing the maximal EMG activation level of the erector spinae for each normalization maneuver. Whiskers show the maximal and minimal values for each test. MVIC, maximal voluntary isometric contraction; FE, flexion-extension; Fsub45, roman chair at 45° with the hips flexed and the trunk parallel to the ground without resistance; Fmax45, roman chair at 45° with the hips flexed and the trunk parallel to the ground with resistance; sub45, roman chair at 45° and the trunk aligned with the lower limbs without resistance; max45, roman chair at 45° and the trunk aligned with the lower limbs with resistance; Hsub, horizontal without resistance; Hmax, horizontal with resistance; B, standing, arms relaxed; B45, standing, arms at 45° with respect to the trunk. a indicates significant differences with respect to FE; b indicates significant differences with respect to Fsub45; c indicates significant differences with respect to Fmax45; d indicates significant differences with respect to sub45; e indicates significant differences with respect to max45; f indicates significant differences with respect to Hsub; g indicates significant differences with respect to Hmax; h indicates significant differences with respect to B; i indicates significant differences with respect to B45.

The ANOVA showed a significant main effect for the type of maneuver (*F*(8, 30) = 315.9, *p* = 8.7 × 10^−27^). None of the three MCIV tests generated the maximal erector spinae activation level in all the participants. The maximal activation level was achieved in 68.4% of cases (26 women) with the test performed with the roman chair in the horizontal position; 26.3% (10 women) achieved maximal activation at 45° with the trunk aligned with the lower extremities and 5.3% (two women) when at 45° with the hips flexed and the trunk parallel to the floor (Fig. 2).

The highest activation percentage (96.3 ± 7.3%; *p* < 0.01 for all the Bonferroni-adjusted paired comparisons with the other eight maneuvers) was with the maximal horizontal test. The test with the roman chair at 45° and the trunk parallel to the floor showed the lowest EMG activation percentage of the three maximal maneuvers (70.1 ± 17%; *p* < 0.01 for the comparisons with the other two maximal maneuvers). Moreover, the percentage of activation achieved in this maximal exercise did not significantly differ from the percentage of EMG activity obtained in the submaximal maneuver with the roman chair in the horizontal position (maximal with roman chair at 45° and trunk parallel to the floor 70.1 ± 17% vs submaximal with roman chair in the horizontal position 61.1 ± 16.7%, *p* = 0.089).

Of the three submaximal maneuvers on the roman chair, the one in the horizontal position produced a significantly higher percentage of activation compared to the other two. The maneuver that resulted in the lowest activation percentage, even lower than trunk flexion-extension, was the maneuver at 45° with the hips flexed and the trunk parallel to the floor (28.5 ± 9.2%; *p* < 0.01 for all the Bonferroni-adjusted paired comparisons with the other eight maneuvers).

The percentage of activation achieved during the submaximal maneuver at 45° with the trunk and lower extremities aligned did not show statistically significant differences with the trunk flexion-extension (43.4 ± 12.0%). In addition, there were no significant differences in the percentage of activity achieved in the trunk flexion-extension exercise compared to the standing position weight-holding test with the arms hanging along the trunk (37.9 ± 9.6%).

The average of the normalized maximum activation peaks of the trunk flexion-extension task exceeded 100% in two cases: normalization as a percentage of the submaximal roman chair maneuver at 45° with the hips flexed and the trunk parallel to the floor (158.6%), and normalization to the standing position weight-holding test with the arms hanging along the trunk (117.0%). The lowest values of inter-individual variability (standard deviation) were achieved when normalizing as a percentage of the standing position weight-holding test at a 45° angle with respect to the trunk (18.2%) and as a percentage of the submaximal roman chair task in the horizontal position (18.9%) (Table 1).

**Table 1:** Average and standard deviation of trunk flexion-extension maximum activation values, normalized as a percentage of each submaximal task. The lowest values of inter-individual variability (standard deviation) are marked in bold.

	**Fsub45**	**sub45**	**Hsub**	**B**	**B45**
** Average**	158.6	98.0	73.3	117.0	83.7
**Standard deviation**	36.5	23.9	**18.9**	26.2	**18.2**

**Notes.**

Fsub45 roman chair at 45° with the hips flexed and the trunk parallel to the ground without resistance sub45 roman chair at 45° and the trunk aligned with the lower limbs without resistance Hsub horizontal without resistance B standing, arms relaxed B45 standing, arms at 45° with respect to the trunk

## Discussion

In this study we performed a battery of 8 different normalization maneuvers (3 maximal tests and 5 submaximal tests) in order to identify which ones produce the highest erector spinae muscle activation in the highest percentage of participants, and the greatest reduction in inter-individual variability. Some of these maneuvers have already been used by other authors to normalize the EMG signal of the lumbar erector spinae, including the trunk extension with resistance exercise (McGill, 1991; Vera-Garcia, Moreside & McGill, 2010; Barbado et al., 2012; García-Vaquero et al., 2012) or the standing position weight-holding test (Lehman, 2002; Callaghan & McGill, 2001). The other maneuvers we used were variations of these two aforementioned exercises. To our knowledge, this is the first time submaximal normalization maneuvers have been studied specifically in the erector spinae.

Of the three MVICs, the results showed that the highest average activation percentage for the erector spinae was obtained when the test was performed in the Biering–Sorensen position. However, this maneuver generated the maximal activation level only in 68.4% of the participants (26 women).

The fact that no single maneuver could generate the maximal level of activation in all the participants has also been observed by other authors for the trunk musculature (Vera-Garcia, Moreside & McGill, 2010) and in other regions such as the shoulder muscles (Dal Maso, Marion & Begon, 2016) and the scapula (Castelein et al., 2015). For this reason, previous studies recommend using several tests instead of only one test to normalize any given muscle (Boettcher, Ginn & Cathers, 2008; Vera-Garcia, Moreside & McGill, 2010; Rutherford, Hubley-Kozey & Stanish, 2011). In our study, around 95% of the participants achieved maximal erector spinae activation when the Biering–Sorensen test was performed either in the horizontal or 45° positions. Based on these results, the use of these two exercises as standardization maneuvers should be considered in future studies, because this would increase the probability of achieving maximal activation in a very high percentage of participants.

It may be that most participants obtained maximal activation in the conventional Biering–Sorensen test, rather than in the 45° inclined position variation, because the flexor moment and subsequent activation demands for the erector spinae are higher in the former. A few authors have also analyzed the EMG activity of the erector spinae on the roman chair at different angles. Specifically, Mayer et al. (1999) analyzed lumbar erector spinae activation while performing a dynamic trunk extension exercise with the roman chair positioned at 6 different angles (0°, 15°, 30°, 45°, 60°, and 75°). Their results showed that the EMG activity of the lumbar paraspinal musculature progressively decreases as the angle to the horizontal increases. Although the type of muscle activation was different between these studies (dynamic versus static), both our results and those of Mayer et al. (1999) indicate that the position of the trunk with respect to the horizontal is an important determinant in achieving the highest degree of erector spinae activation.

In contrast, of the three maximal tests, the exercise with the roman chair at 45° and the trunk parallel to the ground showed the least activation. Compared to the other two tests in which the body is aligned, this test places the trunk at an angular position with respect to the lower extremities. This may cause other extensor muscles such as the hamstrings, gluteus maximus, or thoracic erector spinae to be recruited, which would share the load with the lumbar erector spinae. In addition, when activated, hip extensors are dominant over lumbar extensors, which would decrease further the degree of activation of the erector spinae (Androulakis-Korakakis et al., 2018). In fact, other studies have observed that this oblique position produces less fatigue in the trunk extensor muscles compared to the conventional Biering–Sorensen test (Champagne, Descarreaux & Lafond, 2008), although this must be considered with caution, given the lack of correlation observed between isolated lumbar extensor strength and endurance during the Biering–Sorensen test (Conway et al., 2016). In addition, because the lumbar erector spinae is attached to the pelvis, the angle formed between the trunk and the lower extremities may alter the angle of insertion of the muscle when generating the extensor moment demanded by this posture, which may be also behind the lower levels of activation observed for this test.

Regarding the submaximal normalization tests, the Biering-Sorensen test produced the highest activation levels. Of note, the percentage of activation achieved by this submaximal test was the same as that obtained with the Biering-Sorensen test at 45° with the trunk parallel to the ground against resistance. This result highlights the intense EMG activation this submaximal test can produce in the erector spinae, with values very similar to those achieved by applying resistance to the trunk extension on a roman chair placed at 45° with the trunk parallel to the ground. As in the maximal maneuvers, the submaximal roman chair test that produced the lowest percentage of erector spinae activation was the Biering–Sorensen at 45° with the trunk parallel to the ground. This results suggest that some submaximal maneuvers may activate the muscle as much as some resisted ones. There is a certain degree of variability in the maneuver which will yield the maximum activation levels in each subject, and a non-resisted submaximal one may provide a good reference value, as intense as some of the resisted ones.

Previous studies have used submaximal standing tests to normalize the EMG activity of the erector spinae (Lehman, 2002; Callaghan & McGill, 2001). Lehman (2002) used a submaximal maneuver very similar to the one we used here, except that his participants were asked to maintain a five kg weight, suspended from a chain and a bar, one cm off the ground, and the exact position of their arms was not specified. Callaghan & McGill (2001) also used a standing submaximal maneuver to normalize erector spinae EMG activity; in this case, the participants were asked to hold a 10 kg load with their hands. Again, the authors did not specify the position of the arms. Nonetheless, these studies were investigating static-position tasks not requiring weight (e.g., prolonged sitting without a back support while performing reading tasks, or work at a desk or with a computer), so the activation obtained in the submaximal reference test with weight could easily exceed that produced during these everyday tasks. Thus, it remains unknown whether this test could have been used to normalize more dynamic tasks, such as trunk flexion-extension, which imply more intense musculature activation.

In this study, the flexion-extension movement of the trunk was used as a reference task because it is a very well-studied movement in relation to the appearance of LBP. The only normalization test in which the EMG activity was lower than during the trunk flexion-extension was the submaximal test at 45° with the hips flexed and the trunk parallel to the body. In addition, there were no significant differences between the submaximal standing test in which the participants held two three kg dumbbells with their arms relaxed alongside their body and the flexion-extension reference task. The studies by Lehman (2002) and Callaghan & McGill (2001) do not specify the exact position of the arms, so we do not know if the positions used could also have been used to normalize the trunk flexion-extension. In our study, the positioning of the arms at 45° with respect to the trunk significantly increased the percentage of activation we measured, probably as a response to the increased flexor moment compared to positioning the arms alongside the body.

In any case, our results show that the submaximal test at 45° with the trunk parallel to the floor and the standing test with the arms parallel to the trunk are insufficient to normalize a trunk flexion-extension reference task. In fact, normalized maximum activation peaks of the trunk flexion-extension task exceeded 100% when these two tests were used as reference maneuvers. On the other hand, although the activity produced by the submaximal standing test with the arms at 45° was significantly higher than that obtained during the trunk flexion-extension, the Biering-Sorensen submaximal maneuver without resistance resulted in the highest activation, and also in one of the two lowest inter-individual variability values when used to normalize the reference flexion-extension task.

Other authors have used the non-resisted Biering-Sorensen test to normalize the EMG activity of the erector spinae in pregnant women (Biviá-Roig, Lisón & Sánchez-Zuriaga, 2018; Biviá-Roig, Lisón & Sánchez-Zuriaga, 2019). In this case, the task being studied was muscle activation while standing (Biviá-Roig, Lisón & Sánchez-Zuriaga, 2018) and during trunk flexion-extension (Biviá-Roig, Lisón & Sánchez-Zuriaga, 2019). Given that submaximal maneuvers are designed for use by patients with pain, to avoid injury, and to generate sufficient activation to normalize a wide variety of static and dynamic tasks, we propose using the Biering-Sorensen test without resistance as a submaximal reference test to normalize the erector spinae, more so when the Biering-Sorensen test has been shown repeatedly as a safe maneuver to use in patients with low back pain (Dolan et al., 2000; Conway et al., 2016)

The present study has some limitations. First, we did not analyze the repeatability of the normalization maneuvers. However, previous studies have observed a good repeatability for trunk extensor muscle activation during the Biering–Sorensen test in participants without lumbar pain (Dedering et al., 2000). Second, all the participants in our study were healthy women. We chose these sample parameters to avoid possible variability resulting from gender differences in muscle activation, and to make our results more comparable to previous studies such as Vera-Garcia, Moreside & McGill (2010) which also focused only on women. Although previous studies have observed that muscular responses to different kinds of lumbar extension resistance training are similar in healthy males and females (Fisher et al., 2018), further studies would be required to check the effects of these maneuvers in male volunteers.

## Conclusions

Erector spinae activation patterns have been a target of biomechanical research for a long time, given their relationship to low back pain. Until now there was not a standard submaximal maneuver to normalize its signal, nor all the possible maximal activation maneuvers had been studied. In the present study, a modified Sorensen MVIC test in a horizontal position on a roman chair and against resistance produced the highest erector spinae activation. However, no single maximal test can produce maximal activation in 100% of participants, so the execution of a set of different normalization maneuvers with the trunk at different inclinations should be considered to normalize the erector spinae EMG signal. A modified Sorensen test in a horizontal position and without resistance is the submaximal maneuver that produces the highest erector spinae activation, with values like those obtained in other maneuvers using a roman chair with resistance, and one of the lowest values of inter-individual variability. Therefore, this submaximal modified Sorensen test represents a good reference test to normalize the erector spinae EMG signal, and should be considered as a standard normalization procedure for future studies.

##  Supplemental Information

10.7717/peerj.7824/supp-1 Supplemental Information 1 Percentages of muscle activation in all the subjects for all the tests

## Additional Information and Declarations

### Data Availability

The following information was supplied regarding data availability: The percentages of muscle activation for all the subjects in each of the tests are available in the Supplemental File.
